# How different cardioplegic solutions influence genes expression and cytokine response in an immature rat heart model of ischemia/reperfusion?

**DOI:** 10.1371/journal.pone.0329010

**Published:** 2025-07-29

**Authors:** Arslan Mamedov, Dovydas Gečys, Povilas Jakuška, Rimantas Treinys, Deimantė Narauskaitė, Serik Aitaliyev, Eglė Rumbinaitė, Dainius Karčiauskas, Rimantas Benetis, Edgaras Stankevičius

**Affiliations:** 1 Department of Cardiac, Thoracic and Vascular Surgery, Faculty of Medicine, Medical Academy, Lithuanian University of Health Sciences, Kaunas, Lithuania; 2 Laboratory of Molecular Cardiology, Institute of Cardiology, Medical Academy, Lithuanian University of Health Sciences, Kaunas, Lithuania; 3 Laboratory of Clinical Cardiology, Institute of Cardiology, Medical Academy, Lithuanian University of Health Sciences, Kaunas, Lithuania; 4 Institute of Physiology and Pharmacology, Faculty of Medicine, Medical Academy, Lithuanian University of Health Sciences, Kaunas, Lithuania; 5 Laboratory of Membrane Biophysics, Institute of Cardiology, Medical Academy, Lithuanian University of Health Sciences, Kaunas, Lithuania; 6 Preclinical Research Laboratory for Medicinal Products, Institute of Cardiology, Medical Academy, Lithuanian University of Health Sciences, Kaunas, Lithuania; 7 Faculty of Medicine and Health Care, Al-Farabi Kazakh National University, Almaty, Kazakhstan; 8 Department of Cardiology of Medicine, Medical Academy, Lithuanian University of Health Sciences, Kaunas, Lithuania; Jordan University of Science and Technology Faculty of Medicine, JORDAN

## Abstract

**Introduction:**

The use of cardioplegia not only achieves cardiac arrest but also minimizes ischemic/reperfusion (I/R) injury, potentially improving short- or long-term outcomes. The aim of this study was to evaluate the impact of different cardioplegic solutions – del Nido, Custodiol HTK and St. Thomas on genes expression and cytokines response in an immature rat heart model of I/R using the Langendorff preparation. Expression of genes which are involved in cell cycle, proliferation, apoptosis resistance and response to hypoxia were determined in cardiac tissue, as well as levels pro/anti-inflammatory cytokines were measured.

**Methods:**

A total of 39 male Wistar albino rats were utilized in this study. Experimental animals were divided into 3 groups, four animals in each following groups: St. Thomas (ST), Custodiol HTK (HTK) and del Nido (DN) group. Moreover, each of these groups was divided into 3 groups according to ischemia’s time: 1h ischemia with 20 min reperfusion time, 2h ischemia with 40 min reperfusion time, 4h ischemia with 80 min reperfusion and control groups (K-PRF) with 30 minutes of perfusion was performed in the K-PRF (n = 3). The heart was removed from the chest and immediately frozen at –81°C.

**Results:**

All cardioplegic solutions effectively modulate the expression of HIF1A, FOS, and BNIP2 genes. The results indicated that DN actively induces HIF1A within the first hour. Compared to the ST, and HTK groups, the expression of the HIF1A gene was on average 2 times higher (P < 0.01). Similar results were observed in the 2-hour group. After 4 hours, the effect of cardioplegic solutions continued to maintain the dynamics, but the differences were not statistically significant. The expression of the FOS gene after 2 and 4 hours of incubation with the DN solution remained significantly higher compared to ST (P < 0.05) and HTK (P < 0.05). A comparative analysis with the perfusion group showed that BNIP2 gene expression in the ST and HTK solution groups was significantly lower than in perfused tissue (P < 0.05). Pro-inflammatory cytokines: TNF-alpha, IL-6 and anti-inflammatory cytokines: IL-4 and IL-10 were evaluated. The results showed that there was no statistically significant difference between the groups (P > 0.05).

**Conclusion:**

In our experiment, statistically significant differences were not observed in cytokines. Although statistically significant differences were observed only in gene expression, and only in the rat model, the overall results suggest that del Nido cardioplegic solution may provide better cellular protection. It is also worth mentioning that gene expression and cytokines change are not direct markers of cardioprotection. Further research is needed to confirm these results in human tissues and broader clinical settings.

## Introduction

Ischemia/reperfusion (I/R) injury, which can lead to postoperative myocardial dysfunction, is unavoidable during cardiac surgery. The use of cardioplegia not only achieves cardiac arrest but also minimizes I/R injury, potentially improving short- or long-term outcomes [1].

Prolonged ischemia results in a variety of metabolic and ultrastructural changes in the cardiomyocytes including cell death, which leads to release of fragments of mitochondrial DNA, ATP, and Ca+ into the extracellular space [2–4]. On the other hand, reperfusion of ischemic tissues results in the formation of toxic reactive oxygen species (ROS), including superoxide anions (O2), hydrogen peroxide (H2O2), and nitric oxide-derived peroxynitrite which lead to further vascular cell damage [5].

Based on the sodium-to-potassium ratio, cardioplegic solutions can be categorized into intracellular or extracellular types [6], and the extracellular type is further divided into crystalloid and blood cardioplegia (BC) [7]. Despite profound usage of various cardioplegic solutions, the debate continues regarding which cardioplegia is more effective in preserving myocardial function after I/R injury.

Cardioplegic solutions are essential in cardiac surgery to induce controlled cardiac arrest and protect the myocardium from ischemic damage and ischemia-reperfusion injury [8]. These solutions modulate various cellular pathways, including the expression of genes, which are involved in cellular stress responses and survival mechanisms. Cardioplegic solutions vary significantly in composition, pharmacodynamics, and clinical application. Del Nido cardioplegia, originally developed for pediatric use, contains high potassium and lidocaine, enabling prolonged myocardial arrest with minimal re-dosing [9–11]. St. Thomas solution, rich in potassium and magnesium, is commonly used in adults and requires frequent re-administration [12–14]. Custodiol HTK, a histidine–tryptophan–ketoglutarate-based solution, allows single-dose administration for prolonged procedures by hyperpolarizing the myocyte membrane through sodium and calcium depletion [15–18]. However, comparative data on how these cardioplegic agents modulate molecular responses in immature myocardium remains scarce.

Expanding upon the earlier considerations, our study aimed to simulate the clinical context and evaluate the impact of del Nido, Custodiol HTK, and St. Thomas solutions on cardiac function in an immature rat model of I/R using the Langendorff preparation. Cardioplegic solutions were evaluated based on their cardiac – protective properties. For such analysis, expression of genes which are involved in cell cycle, proliferation, apoptosis resistance and response to hypoxia were determined in cardiac tissue, as well as levels pro/anti-inflammatory cytokines were measured.

The HIF1A gene plays a key role in helping cells adapt to low-oxygen conditions commonly encountered during cardiac surgery by promoting oxygen delivery and metabolic adjustments [19]. The FOS gene, a component of the AP-1 transcription factor complex, supports cellular responses such as proliferation and survival under surgical stress [20]. Meanwhile, BNIP2 contributes to the regulation of apoptosis, and its control may be essential for reducing cardiac cell loss and preserving heart function during ischemic events [21].

## Materials and methods

### Animal model

The pilot study was carried out on 41 males Wistar rats, 1 month (90 ± 30 body weight), which were purchased from the Lithuanian University of Health Sciences Veterinary Academy (Kaunas, Lithuania). The rats were housed under controlled conditions with a 12-hour light/dark cycle and given access to food and water ad libitum. All experiments were performed in accordance with the Guide for the Care and Use of Laboratory Animals published by the US National Institutes of Health (NIH Publication No. 85–23, revised 1996), and 3Rs principle proposed by Russell and Burch (1959). Experiments were approved by the State Food and Veterinary Service of the Republic of Lithuania Number of approval: Nr. G2-265).

**Fig 1:**
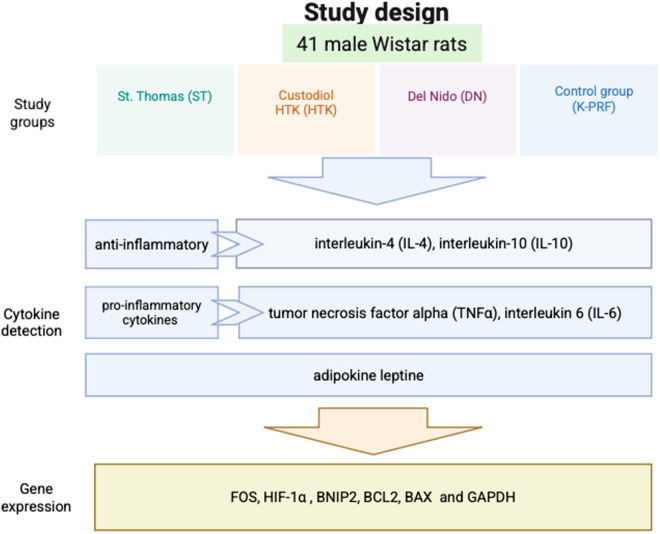
Study design.

Animals were assigned into 3 groups according to cardioplegic treatment, four animals in each following groups: ST. Thomas (ST), Custodiol HTK (HTK) and del Nido (DN). Moreover, each of these groups were further divided into 3 sub-groups based on ischemia’s time: (i) 1 h ischemia + 20 min reperfusion; (ii) 2 h ischemia + 40 min reperfusion; (iii) 4 h ischemia + 80 min reperfusion; (iv) control group (K-PRF) no myocardial ischemia + 30 min perfusion (n = 5). The heart was removed from the chest and immediately frozen at –81°C. The schematic study design is depicted in Fig 1.

### Heart excision and incubation

Rats were sacrificed following standard animal experimentation protocols. Anesthesia was induced using Ketamine (90 mg/kg) and Xylazine (9 mg/kg). The disappearance of the pedal pain withdrawal reflex indicated deep anesthesia. Subcutaneous administration of heparin (5 IU/g body weight) occurred 10 minutes before cervical dislocation. A skin incision was made at the xyphoid-sternum. The anterior chest was deflected upward, the thymus was excised, and the pericardium was opened. Finally, the aorta, superior vena cava, and inferior vena cava were isolated.

After the heart was removed, it was immediately transferred to a dissection dish containing cold perfusion buffer. The aorta was gently cannulated (to avoid aortic valve damage) and ligated using double 5−0 silk sutures. In our study, we employed the retrograde heart perfusion model described by O. Langendorff [22,23]. The heart was mounted on the Langendorff system within 5 minutes. Subsequently, the heart was perfused with buffer for 15 minutes in each case. After continuous perfusion for 15 minutes, cardiac function nearly reached a physiological state. The heart was perfused in constant flow mode, with careful attention to preventing air embolism. For perfusion step Modified Krebs-Henseleit buffer (NaCl 135.0 mM, MgCl_2_ 0,9 mM, NaH_2_PO_4_ 0,33 mM, KCl 5,4 mM, CaCl_2_ 1.0 mM, glucose 10 mM, HEPES 10 mM) prepared in deionized water was used. Perfusion buffer was oxygenated through bubbling 95% 0_2_.

Coronary flow was measured before cardioplegia by assessing the effluent volume over a 2-minute period. Throughout the experiment, we strictly monitored and regulated the temperature of the water-jacketed reservoir and perfusion buffer. The heat exchanger maintained normothermia at +37°C, while the cardioplegic solutions were kept at a temperature of +4°C.

### Cardioplegia solution infusion

Approximately 10 ml of cold (+4°C) cardioplegia solution (ST, HTK or DN), was infused into the aorta. An infusion of cardioplegic solution was carried out for 2 minutes. When we infused cardioplegic solution a heart was stopped and ischemic time begging. Re-plegia (redosing) were performed every 30 min in the ST cardioplegic solution groups and every 90 min in the DN groups. In the HTK groups, only a single dose of cardioplegia was used. Re-plegia (redosing) also carried out for 2 minutes. For the DN solution, heparinized rat blood was drawn from the rat’s tail and mixed at a ratio of 4:1 (4 parts crystalloid to 1 part blood). The time before asystole was recorded. After the experiment, entire left ventricular were harvested and stored at −80°C until analysis.

### Cytokine detection assay

In this study, we employed the Luminex (Luminex Corporation, Austin, TX, USA) multiplex cytokine assay to quantitatively analyze pro-inflammatory and anti-inflammatory cytokines in immature rat heart tissue. Initially, heart tissues were collected and promptly processed to minimize degradation. Heart tissues were ground up and homogenized using liquid nitrogen. Each sample was weighed and placed in a 2 mL microcentrifuge tube. Following weighting, 500 µL of Cell Lysis Buffer for every 100 mg of tissue, facilitating the breakdown of cellular structures and the release of intracellular components. Homogenization was performed using a TissueLyser operating at 25 Hz for 2 min which was determined to be the optimal time for achieving a uniform homogenate without overheating the sample. After homogenization, the samples were centrifuged at 16,000 × g for 10 min at 4°C. The clear supernatant was transferred to a new tube and total protein concentrations were measured using the Bio-Rad™ DC Protein Assay Kit.

Levels of anti-inflammatory (interleukin-4 (IL-4), interleukin-10 (IL-10)), adipokine leptine and pro-inflammatory cytokines (tumor necrosis factor alpha (TNFα), interleukin 6 (IL-6), were quantified using Rat Custom ProcartaPlex Mix&Match 5-Plex Kit (Thermo Fisher Scientific, Austria), following the manufacturer’s instruction. Samples were analyzed in duplicates. Data analysis utilized the Luminex xPONENT software, calculating cytokine concentrations based on standard curves established for each cytokine.

### Real-time qPCR

To better define the differences between cardioplegic solutions, real-time qPCR was used to conduct gene expression analysis to evaluate apoptosis, hypoxia, and proliferation in heart tissue cells. HIF1A gene is crucial for cellular response to hypoxia, a common condition during cardiac surgery when blood supply is limited. HIF1A activates pathways that help cells survive under stress, like promoting oxygen delivery and metabolism adaptations [24]. FOS is part of the AP-1 transcription factor complex, which regulates cell proliferation, differentiation, and survival, especially under stress [20]. Its modulation helps the heart tissue manage the surgical and ischemic stresses introduced during procedures. BNIP2 is involved in apoptosis [25,26]. During cardiac surgery, regulating cell death is crucial to preserve heart function, and the modulation of BNIP2 could help minimize unwanted cell death.

Heart tissues were homogenized using liquid nitrogen as described below. Samples were immediately lysed with TRIzol^TM^ (Invitrogen, Netherlands) reagent. Total RNA was extracted using commercial PureLink^TM^ RNA Mini Kit (Invitrogen, Netherlands) according to manufacturers’ manual. RNA concentration and purity were evaluated by NanoDrop 1000 spectrophotometer (Thermo Fisher Scientific, Netherlands). Reverse transcription was performed using High-Capacity RNA-to-cDNA^TM^ kit (Applied Biosystems, USA). Real-time qPCR was performed using TaqMan^TM^ Gene Expression methodology (Applied Biosystems, USA) according to manufacturers’ protocol. Probes and primers for FOS (Rn02396759_m1), HIF-1α (Rn01472831_m1), BNIP2 (Rn01530716_m1), BCL2 (Rn99999125_m1), BAX (Rn01480161_g1) and GAPDH (Rn01775763_g1) were used. Gene expression data was normalized to GAPDH gene and calculated using 2^-ΔΔCT^ method [27].

### Statistical analysis

Study sample size was determined by evaluating Cohen’s d coefficient. Based on the size effect, minimal number of animals was chosen for each condition (n = 4). Data were expressed as mean values ± standard deviation. Normality of data was evaluated by Shapiro-Wilk test.

Given the experimental design – including three cardioplegic solutions (St. Thomas, Custodiol HTK, del Nido) and three ischemia durations (1, 2, and 4 hours) – multi-factorial analyses were performed. Specifically, two-way ANOVA was used to assess the main effects of cardioplegic solution and ischemia duration, as well as their interaction, on gene expression (HIF1α, FOS, BNIP2, BAX/BCL2) and cytokine concentrations (IL-4, IL-6, IL-10, leptin, TNF-α). Where appropriate, post hoc comparisons were conducted to determine differences between groups. The homogeneity of variances was evaluated by Levene’s test. If variances were unequal, this was noted in the results, and findings interpreted with caution.

A p-value of less than 0.05 was considered statistically significant. All analyses were performed using SPSS, v22.0 and visualized using GraphPad Prism 9 software.

## Results

### Effects of cardioplegic solutions on cellular resistance to apoptotic stimuli

**Fig 2:**
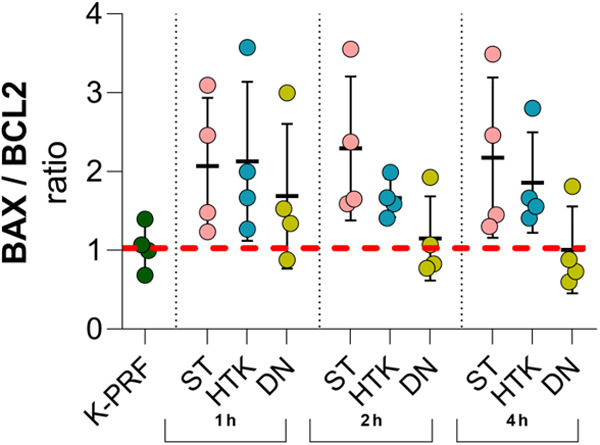
The ratio of BAX and BCL2 gene expression in rat heart tissues (n = 4) at different time intervals. The relative gene expression study of heart tissues treated with cardioplegic solutions showed that DN has the strongest anti-apoptotic effect, which becomes evident at 2 h. The results also indicate that the effect of DN on heart tissues is similar to perfusion. Despite quantitative differences, these results were not statistically significant (2 h: DN vs. ST (P = 0.08) and HTK (P = 0.12); 4 h: DN vs. ST (P = 0.07) and HTK (P = 0.09), Student’s T-test).

Ischemia-reperfusion (I/R) injury leads to cell death primarily due to the sudden restoration of blood flow and oxygen to previously oxygen-deprived tissues, which generates excessive reactive oxygen species (ROS) and triggers inflammatory responses, ultimately causing significant cellular damage and death [28]. To evaluate cell susceptibility to apoptotic stimuli during cardiac intervention using different CP solutions, expression of genes, coding BCL2 apoptosis regulator (BCL2) and BCL2 associated X, apoptosis regulator (BAX) was evaluated. The ratio of BAX to BCL2 gene expression can be used as an indicator to the cells’ response to apoptotic stimuli: an increased BAX/BCL2 ratio reduces the cells’ resistance to apoptotic stimuli. These genes are closely related and act as pro-apoptotic and anti-apoptotic regulators, respectively [29]. The results indicated that the BAX/BCL2 ratio in tissue treated with DN solution was approximately 50% lower compared to ST and HTK solutions, however these findings did not yield statistical significance (Fig 2). However, it can be speculated that DN might induce cellular resistance to apoptotic stimuli, thus providing superior cardioprotective properties when compared to ST and HTK.

### Contrasting potency of cardioplegic solutions in cellular processes

To further investigate CP effects on cellular processes in cardiac tissue, expression of genes coding BCL2 interacting protein (BNIP2), FOS proto-oncogene (FOS) and hypoxia inducible factor 1 subunit alpha (HIF1α) were evaluated (Table 1).

**Table 1:** Relative gene expression data. Exact p values are given (Students p test).

1 h		HIF1A			FOS			BNIP2		
Group	N	Mean ∆CT ± SD	Mean Fold Change ± SD	P value	Mean ∆CT ± SD	Mean Fold Change ± SD	P value	Mean ∆CT ± SD	Mean Fold Change ± SD	P value
ST	4	2.36 ± 0.22	0.58 ± 0.09	^a^**0.007** ^b^0.195 ^c^**0.002**	−4.59 ± 0.22	0.49 ± 0.07	^a^**0.000** ^b^0.292 ^c^0.287	1.38 ± 0.21	0.68 ± 0.40	^a^**0.000** ^b^0.598 ^c^0.094
HTK	4	2.18 ± 0.11	0.65 ± 0.05	^a^ **0.011** ^c^ **0.003**	−4.28 ± 0.50	0.41 ± 0.14	^a^**0.002** ^c^0.117	1.47 ± 0.24	0.51 ± 0.09	^a^**0.000** ^c^0.074
DN	4	1.41 ± 0.32	1.14 ± 0.25	^a^0.532	−5.38 ± 1.33	1.13 ± 1.01	^a^0.699	0.48 ± 0.88	1.07 ± 0.77	^a^0.784
**2 h**		**HIF1A**			**FOS**			**BNIP2**		
**Group**	**N**	Mean ∆CT ± SD	Mean Fold Change ± SD	P value	Mean ∆CT ± SD	Mean Fold Change ± SD	P value	Mean ∆CT ± SD	Mean Fold Change ± SD	P value
ST	4	2.22 ± 0.22	0.64 ± 0.07	^a^**0.015** ^b^**0.002** ^c^**0.0008**	−4.60 ± 0.55	0.51 ± 0.2	^a^**0.008** ^b^0.935 ^c^**0.017**	1.51 ± 0.16	0.45 ± 0.05	^a^ **0.000** ^b^ **0.013** ^c^ **0.005**
HTK	4	1.64 ± 0.16	0.96 ± 0.1	^a^0.728 ^c^**0.028**	−4.64 ± 0.93	0.56 ± 0.26	^a^**0.024** ^c^**0.025**	0.96 ± 0.27	0.68 ± 0.11	^a^**0.009** ^c^0.085
DN	4	1.13 ± 0.31	1.38 ± 0.3	^a^0.102	−6.93 ± 0.86	2.75 ± 1.4	^a^ **0.024**	0.37 ± 0.51	1.03 ± 0.35	^a^0.928
**4 h**		**HIF1A**			**FOS**			**BNIP2**		
Group	N	Mean ∆CT ± SD	Mean Fold Change ± SD	P value	Mean ∆CT ± SD	Mean Fold Change ± SD	P value	Mean ∆CT ± SD	Mean Fold Change ± SD	P value
ST	4	2.29 ± 0.31	0.62 ± 0.12	^a^**0.018**^b^0.092 ^c^0.102	−4.80 ± 0.42	0.60 ± 0.15	^a^**0.007** ^b^0.815 ^c^**0.032**	1.82 ± 0.54	0.42 ± 0.13	^a^**0.002** ^b^0.153 ^c^0.108
HTK	4	1.84 ± 0.34	0.85 ± 0.19	^a^0.291 ^c^0.456	−4.93 ± 1.03	0.73 ± 0.47	^a^0.215 ^c^0.088	1.18 ± 0.56	0.66 ± 0.25	^a^**0.030** ^c^0.479
DN	4	1.52 ± 0.74	1.14 ± 0.55	^a^0.904	−6.52 ± 1.17	2.32 ± 1.8	^a^0.187	0.75 ± 1.1	0.90 ± 0.54	^a^0.462
		**HIF1A**			**FOS**			**BNIP2**		
Group	N	Mean ∆CT ± SD	Mean Fold Change ± SD	P value	Mean ∆CT ± SD	Mean Fold Change ± SD	P value	Mean ∆CT ± SD	Mean Fold Change ± SD	P value
K-PRF	5	1.57 ± 0.33	–	–	−5.64 ± 0.05	–	–	0.35 ± 19	**–**	**–**

**Fig 3:**
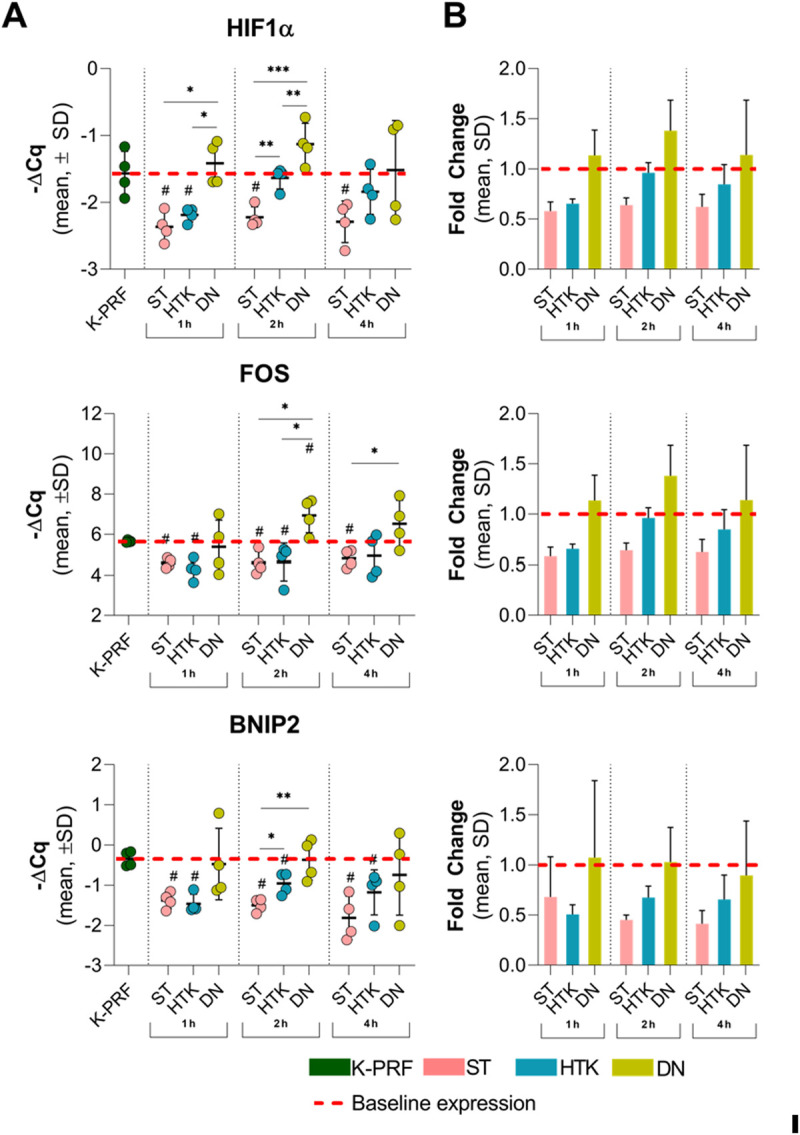
Results of the real-time PCR study for HIF1A, FOS, and BNIP2. The figure (**A**) represents -∆Ct values normalized to the reference gene (GAPDH). In all study groups, the DN solution induced the highest gene expression, comparable to the perfusion procedure. The most statistically significant differences were observed after 2 h of incubation. # compared to perfusion P < 0.05; * P < 0.05; ** P < 0.01; *** P < 0.001; Student’s t-test. The next panel (**B**) depicts average fold change values normalized to perfusion group. In all groups the DN treatment resulted in similar or upregulated expressions of HIF1A, FOS, and BNIP2 genes compared to perfused heart tissue.

It is known that BNIP2 is important for skeletal muscle differentiation as well as in myosin II-dependent contractility where BNIP2 knockdown results in reduced recoil velocity of actin filaments [30]. Our data indicate that treatment with DN solution retains relative BNIP2 mRNA levels, whereas ST and HTK solutions provide inferior effects (Fig 3A) and are significantly (P < 0.05) reduced to approximately 70%, when compared to perfused heart tissue in all time stamps. However, this effect of DN was only observed in the first 2 h of the experiment, thus gradually depleting. Overall, the data shows that DN could be beneficial for the maintenance of cardiomyocyte contractile function.

In addition to BNIP2, we have evaluated the expression of HIF-1α mRNA. HIF-1α plays a critical role in adaptive responses to myocardial ischemia by promoting formation of collateral arteries and inhibit cardiomyocyte death [31]. Studies show that during I/R, HIF-1α alleviates ROS production by upregulating glycolytic enzymes and pyruvate dehydrogenase kinase 1 (PDK1), which limits the influx of substrates into the mitochondria [32]. Our results demonstrate that treatment DN effectively induces HIF1α mRNA (Fig 3B). Significant changes were observed in 1 h and 2 h timestamps and, the effects were depleted after 4 h. When compared to ST and HTK solutions, DN exerted a 90% higher (P < 0.01) relative HIF1α expression, thus providing additional evidence on DN cardioprotective properties.

FOS is a member of FOS family proteins, which dimerize with proteins of the JUN family, resulting in the transcription factor complex activation protein 1 (AP-1). AP-1 regulate a range of cellular and biological processes, including cell cycle, proliferation, programmed cell death and others [33]. Our data show, that relative c-FOS mRNA levels remained significantly higher in DN group compared to ST (P < 0.05) and HTK (P < 0.05). Expression of the FOS gene after exposure to DN was similar to that of perfused tissue in the 1 and 4-hour groups, while in the 2-hour group it was significantly higher (P < 0.05). The results significantly differed when comparing the FOS gene expression in heart tissues treated with ST and HTK solutions to perfused tissue – the FOS gene expression in cells treated with these cardioplegic solutions was significantly (P < 0.05) lower than in perfused tissue (Fig 3C).

To summarize, gene expression data represents that DN solution have significantly stronger cardioprotective properties, when compared to ST and DN. In addition, all results involving DN were comparable to perfused tissue, which supplements the aforementioned conclusion.

### Gene expression response to cardioplegic solutions and ischemia duration

Analysis of myocardial gene expression revealed significant differences between cardioplegic solutions, particularly for markers of hypoxia and cellular stress (Supplements S1-S4 Tables ). As shown in S1 Table, two-way ANOVA demonstrated a significant main effect of solution on HIF1α expression at both 1 hour (p = 0.007) and 2 hours (p = 0.015), with post hoc analysis indicating that the DN group exhibited significantly lower ΔCt values (reflecting higher expression) compared to ST and HTK. The highest HIF1α expression was observed in DN at 2 hours (1.13 ± 0.31), while the highest ΔCt values (indicating lowest expression) were noted in ST at 1 hour (2.36 ± 0.22). By 4 hours, differences between groups were less pronounced and not statistically significant (p > 0.05).

Similarly, FOS gene expression (S2 Table) differed significantly between groups at 2 hours, with DN showing the lowest ΔCt values (highest expression; −6.93 ± 0.86) compared to ST (−4.60 ± 0.55; p = 0.008) and HTK (−4.64 ± 0.93; p = 0.024). Although FOS expression was also numerically higher in DN at 1 hour and at 4 hours (−6.52 ± 1.17), these differences were not statistically significant at those time points.

BNIP2 gene expression patterns (S3 Table) mirrored these findings, with significantly higher expression in the DN at both 1 hour (ΔCt 0.48 ± 0.88) and 2 hours (0.37 ± 0.51) compared to ST (1 h: 1.38 ± 0.21, p < 0.001; 2 h: 1.51 ± 0.16, p = 0.005) and HTK (1 h: 1.47 ± 0.24, p < 0.001). At 2 hours, the difference between DN and HTK did not reach statistical significance (p = 0.085), and by 4 hours, no significant group differences were detected.

Finally, the BAX/BCL2 ratio (S4 Table), a marker of cellular susceptibility to apoptosis, tended to be lower in the DN at 2 hours (0.46 ± 0.10) and 4 hours (0.65 ± 0.17) relative to ST (2 h: 0.80 ± 0.15, 4 h: 0.94 ± 0.12) and HTK (2 h: 0.89 ± 0.17, 4 h: 0.94 ± 0.14); however, these differences did not reach statistical significance (all p > 0.05).

### Effects of cardioplegic solution on cytokine expression

**Fig 4:**
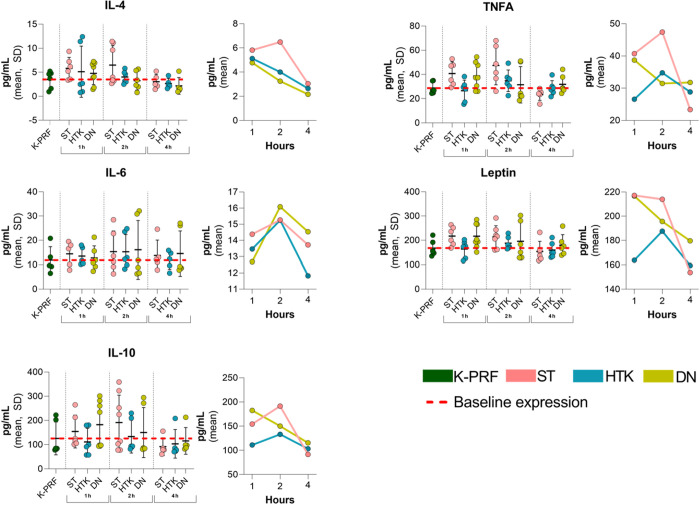
Results of cytokine expression. Despite quantitative differences between groups, these results were not statistically significant (P > 0.05).

Inflammatory cytokines are one of the major indices in myocardial animal models. To determine which cardioplegic solution better preserves immature rat myocardium, pro-inflammatory cytokines TNF-alpha and IL-6 were evaluated. The results showed (Fig 4) that there was no statistically significant difference between the groups (P > 0.05). However, under conditions of prolonged ischemia, inflammation was reduced the most in the HTK group.

Further evaluation was conducted on anti-inflammatory cytokines: IL-4 and IL-10. Although there was no statistically significant difference between the groups (Fig 4), we noticed, that the number of anti-inflammatory cytokines decreases over time in all cardioplegic groups.

When assessing the effect of ischemia-reperfusion on leptin, it was noted that no significant difference was found between cardioplegic solutions (P > 0.05). However, in the 4-hour ischemia group, the level of leptin decreased in all groups (Fig 4).

### Cytokine profile across experimental groups

Analysis of cytokine concentrations across cardioplegic solutions and ischemia durations revealed no significant differences for any measured marker (Supplement S5-S9 Tables).

IL-4 levels are summarized in S5 Table . Two-way ANOVA demonstrated no significant main effect of solution or ischemia duration, nor interaction effect, on IL-4 concentration (F (8.48) = 0.81, p = 0.599, partial η² = 0.13). The highest mean IL-4 was seen in ST at 2 hours (7.36 ± 4.48 pg/mL), and the lowest in DN at 4 hours (2.78 ± 2.16 pg/mL). Similarly, as shown in S6 Table , IL-6 concentrations did not vary significantly by solution or ischemia duration (all p > 0.05), with means ranging from 24.09 ± 18.22 pg/mL (ST, 2 h) to 14.53 ± 9.29 pg/mL (DN, 4 h), and Levene’s test indicating homogeneity of variances (p = 0.092).

For IL-10 (S7 Table), two-way ANOVA again showed no significant effects of solution, ischemia duration, or their interaction (all p > 0.05), with mean values from 49.45 ± 16.55 pg/mL (ST, 4 h) to 58.41 ± 22.41 pg/mL (DN, 1 h); Levene’s test confirmed variance homogeneity (p = 0.289).

Leptin concentrations (S8 Table) also did not differ significantly between solutions or durations (all p > 0.05), with the highest value in ST at 1 hour (25.20 ± 6.73 pg/mL) and the lowest in DN at 4 hours (17.37 ± 5.33 pg/mL), and Levene’s test supporting homogeneity of variance (p = 0.545).

TNF-α concentrations by cardioplegic solution and ischemia duration are presented in S9 Table. Two-way ANOVA found no significant main effects of solution or ischemia duration, and no significant interaction, for myocardial TNF-α levels (all p > 0.05). Mean TNF-α ranged from 6.46 ± 2.20 pg/mL (ST at 4 h) to 7.84 ± 1.80 pg/mL (HTK at 1 h), with no statistically significant differences detected. Levene’s test confirmed homogeneity of variances (p = 0.877).

Collectively, these findings indicate that, within the studied range, neither the choice of cardioplegic solution nor the duration of ischemia had a statistically significant impact on the myocardial release of IL-4, IL-6, IL-10, leptin, TNF-α following ischemia/reperfusion injury.

## Discussion

Cardioplegia solutions such as Del Nido, St. Thomas, and Custodiol have different time frames and infusion rates due to their unique compositions, pharmacodynamics, and intended applications.

The extracellular del Nido cardioplegia solution was first formulated for pediatric cardiac surgery in the early 1990s and has been widely used ever since [9,34]. Del Nido is high in potassium and contains lidocaine for membrane stabilization, reducing the need for frequent dosing. It can provide myocardial arrest with a single dose that lasts up to 90 min [11].

St. Thomas solution is a simpler cardioplegia formula with potassium and magnesium, commonly used in adult heart surgeries. It typically requires re-administration every 20–30 min to maintain myocardial protection, making it ideal for procedures that need intermittent doses [12]. Both, del Nido and St. Thomas solutions, contributes to maintaining the cell membrane depolarization phase [14,35].

The histidine–tryptophan–ketoglutarate (often called Custodiol HTK) solution was developed by Bretschneider in 1964 and has been used for several decades as one of the preservation methods in heart surgery.

By combining metabolic suppression, pH buffering, membrane stabilization, and provision of an energy substrate, HTK solution protects the myocardium for up to 4 hours, which is often sufficient for complex cardiac surgeries [15,16]. It is identified as an intracellular, crystalloid cardioplegia because of its low levels of sodium and calcium. Sodium depletion of the extracellular space causes a hyperpolarization of the myocyte plasma membrane, inducing cardiac arrest in diastoles [17,18].

Based on gene expression and cytokine analysis, in our study del Nido showed better myocardium preservation compared to St Thomas and Custodiol HTK. Similarly, del Nido outperformed other cardioplegic solutions in terms of stress/heat shock proteins, which are indicators of cellular damage in infant’s myocardium [19]. Moreover, blood cardioplegia (del Nido) more effective to stimulate stress/heat shock protein 70−1 gene expression compared to crystalloid cardioplegic solutions (St Thomas and Custodiol HTK) in pediatric population [36].

While specific studies directly linking cardioplegic solutions to the modulation of these genes are limited, the protective effects of these solutions on myocardial tissue suggest an influence on pathways involving HIF-1α, FOS, and BNIP2. An early in vitro study has observed that adenovirus-mediated overexpression of HIF-1α protected cultured neonatal cardiomyocytes against ischemia-reperfusion injury [37]. Also, it is important to emphasize that HIF-1α plays an important role as cell proliferation enhancer. Evidence shows that HIF1-α can repress cellular stress to allow sustain proliferation of hypoxic fetal cardiomyocytes [38], as well as inhibition of HIF1-α results in reduced glucose uptake in cardiomyocytes following I/R injury in animals, proving its essential role in maintaining homeostasis in heart tissue [39]. Despite being essential for protective mitochondrial function, studies focusing on BNIP2 are scarce. Based on available literature, BNIP2 expression homeostasis is important for contractility maintenance and myogenic phenotype upkeep [30,40]. Nevertheless, a recent study observed a close interaction between HIF1-α and BNIP3 gene. Based in the results, HIF-1α enhances BNIP3 expression during oxygen-glucose deprivation/recovery injury, which induces autophagy in cardiomyoblasts *in vitro,* resulting in increased cell survival [41]. Furthermore, an early study indicated that deficiency in c-FOS increases infarct size and myocardial apoptosis leading to impaired cardiac function post-infarction [42], however another study implicated that c-FOS could regulate miR-27a which contributes to myocardial ischemia-reperfusion injury by regulated the translocation of apoptosis-inducing factor from the mitochondria to the nucleus [43].

Despite our findings, further research is needed to elucidate the exact molecular mechanisms by which cardioplegic solutions affect these gene expressions.

### Leptin

The adipokine leptin, derived from adipocytes, plays a crucial role in regulating energy balance, cell metabolism, inflammatory and immune responses, and cardiovascular homeostasis. Leptin resistance, common in obese or type 2 diabetes mellitus patients, is characterized by a reduced tissue response to leptin [44].

In the cardiovascular system, this resistance negatively impacts the heart’s stress response, promoting cardiac remodeling through impaired metabolism, increased fibrosis, vascular dysfunction, and heightened inflammation [44].

Although elevated circulating leptin levels have been linked to an increased cardiovascular risk in humans. However, recent meta-analyses indicate that some epidemiological studies did not find this association. Studies conducted on mice deficient in leptin or leptin receptors often yield contradictory results, showing both protective and harmful effects of leptin [45].

Additionally, mouse models have a significantly different lipoprotein metabolism compared to humans, which limits the extrapolation of these results to human physiology [45].

Our results indicate that all cardioplegic solutions reduce leptin levels following I/R, particularly in cases of prolonged myocardial ischemia. A decrease in leptin levels may indicate that changes in cardiomyocyte proteins are occurring over time. Although we didn’t find any significant differences between groups.

### Cytokines: *TNF-alfa, IL-6 and IL-4, IL-10*

Although there is no significant difference between the cardioplegia groups, the trend shows that the results for HTK and DN are closer to those of K-PRF (myocardial perfusion without ischemia). Interestingly, the indicators are highest in the 2-hour group, which could be due to a greater early inflammatory reaction to I/R. In research conducted by Gorjipour et al., two cardioplegic solutions, del Nido and modified St. Thomas, were compared. The authors noted that no significant differences were found in the TNF-alpha and IL-6 groups, but all cytokines showed significant increases after cardiac surgery [46].

We noticed that the number of anti-inflammatory cytokines decreases over time in all cardioplegic groups. Large decreases are observed in IL-4 groups.

For IL-10, the number of anti-inflammatory cytokines remains more stable, although it decreases over time, and the results are more closely associated with K-PRF. In research conducted by Gorjipour et al., it was observed that the anti-inflammatory cytokine response in the modified St. Thomas, group is significantly better than in the del Nido group. IL-10 levels showed a moderately significant increase in the modified ST. Thomas group compared to the del Nido group after surgery. The authors noted that this could be due to shorter intervals of the modified St. Thomas cardioplegia solution administration, which prevents rewarming of the myocardium, increased metabolic demand and hypoxia [46].

Cytokine analysis revealed that, in our case, anti-inflammatory cytokines more accurately reflect the initial changes in the immature myocardium under ischemic conditions. However, there is no doubt that further, broader studies are needed to observe more pronounced differences.

## Conclusion

In our experiment, statistically significant differences were not observed in cytokines. Although statistically significant differences were observed only in gene expression, and only in the rat model, the overall results suggest that del Nido cardioplegic solution may provide better cellular protection. It is also worth mentioning that gene expression and cytokines change are not direct markers of cardioprotection. Further research is needed to confirm these results in human tissues and broader clinical settings.

## Study limitations

This study has several limitations. The use of Wistar rats as an animal model and the focus on short-term parameters limit its direct application to pediatric cardiac surgery in humans. Additionally, the lack of long-term observation under circumstances where some changes currently observed might become more noticeable. The small sample size of our study indicates possible data variability; therefore, a larger sample study is needed to verify the reliability of the obtained data. Further research is needed to confirm these results in human tissues and broader clinical settings.

## Supporting information

S1 Table HIF1α ΔCt by solution and ischemia duration. (PDF)

S2 Table FOS ΔCt by solution and ischemia duration. (PDF)

S3 Table BNIP2 ΔCt by solution and ischemia duration. (PDF)

S4 Table BAX/BCL2 Ratio by solution and ischemia duration. (PDF)

S5 Table IL-4 Levels by Solution and Ischemia Duration. (PDF)

S6 Table IL-6 levels by solution and ischemia duration. (PDF)

S7 Table IL-10 levels by solution and ischemia duration. (PDF)

S8 Table Leptin levels by solution and ischemia duration. (PDF)

S9 Table TNF-α levels by solution and ischemia duration. (PDF)

## References

[1] Ghimire A, Bisset ES, Howlett SE. Ischemia and reperfusion injury following cardioplegic arrest is attenuated by age and testosterone deficiency in male but not female mice. Biol Sex Differ. 2019;10(1):42. doi: 10.1186/s13293-019-0256-4 31443710 PMC6708213

[2] Monassier JP. Reperfusion injury in acute myocardial infarction. From bench to cath lab. Part I: Basic considerations. Arch Cardiovasc Dis. 2008;101(7–8):491–500. doi: 10.1016/j.acvd.2008.06.014 18848692

[3] Sánchez-Hernández CD, Torres-Alarcón LA, González-Cortés A, Peón AN. Ischemia/Reperfusion Injury: Pathophysiology, Current Clinical Management, and Potential Preventive Approaches. Mediators Inflamm. 2020;2020:8405370. doi: 10.1155/2020/8405370 32410868 PMC7204323

[4] Wang S, Li Y, Song X, Wang X, Zhao C, Chen A, et al. Febuxostat pretreatment attenuates myocardial ischemia/reperfusion injury via mitochondrial apoptosis. J Transl Med. 2015;13:209. doi: 10.1186/s12967-015-0578-x 26136232 PMC4489215

[5] Collard CD, Gelman S. Pathophysiology, Clinical Manifestations, and Prevention of Ischemia-Reperfusion Injury. Anesthesiology. 2001;94(6):1133–8. doi: 10.1097/00000542-200106000-00030 11465607

[6] Michel P, Vial R, Rodriguez C, Ferrera R. A comparative study of the most widely used solutions for cardiac graft preservation during hypothermia. J Heart Lung Transplant. 2002;21(9):1030–9. doi: 10.1016/s1053-2498(02)00414-x 12231375

[7] Boening A, Hinke M, Heep M, Boengler K, Niemann B, Grieshaber P. Cardiac surgery in acute myocardial infarction: crystalloid versus blood cardioplegia – an experimental study. J Cardiothorac Surg. 2020;15(1). doi: 10.1186/s13019-020-1058-9 PMC6950911 31915024

[8] Glöckner A, Ossmann S, Ginther A, Kang J, Borger MA, Hoyer A, et al. Relevance and Recommendations for the Application of Cardioplegic Solutions in Cardiopulmonary Bypass Surgery in Pigs. Biomedicines. 2021;9(9):1279. doi: 10.3390/biomedicines9091279 34572465 PMC8464907

[9] Matte GS, del Nido PJ. History and Use of del Nido Cardioplegia Solution at Boston Children’s Hospital. J Extra Corpor Technol. 2012;44(3):98–103. doi: 10.1051/ject/201244098 23198389 PMC4557532

[10] Salameh A, Dhein S. Strategies for Pharmacological Organoprotection during Extracorporeal Circulation Targeting Ischemia-Reperfusion Injury. Front Pharmacol. 2015;6:296. doi: 10.3389/fphar.2015.00296 26733868 PMC4686733

[11] Waterford SD, Ad N. Del Nido cardioplegia: Questions and (some) answers. J Thorac Cardiovasc Surg. 2023;165(3):1104–8. doi: 10.1016/j.jtcvs.2021.11.053 35074182

[12] Ibrahim MF, Venn GE, Young CP, Chambers DJ. A clinical comparative study between crystalloid and blood-based St Thomas’ hospital cardioplegic solution. Eur J Cardiothorac Surg. 1999;15(1):75. 10077377 10.1016/s1010-7940(98)00287-5

[13] Mishra P, Jadhav RB, Mohapatra CKR, Khandekar J, Raut C, Ammannaya GK, et al. Comparison of del Nido cardioplegia and St. Thomas Hospital solution – two types of cardioplegia in adult cardiac surgery. kitp. 2016;4:295–9. doi: 10.5114/kitp.2016.64867 PMC5233756 28096823

[14] Caputo M, Modi P, Imura H, Pawade A, Parry AJ, Suleiman MS. Cold blood versus cold crystalloid cardioplegia for repair of ventricular septal defects in pediatric heart surgery: a randomized controlled trial. 2002. 10.1016/s0003-4975(02)03695-0 12173840

[15] Mercan I, Dereli Y, Topcu C, Tanyeli O, Isik M, Gormus N, et al. Comparison between the Effects of Bretschneider’s HTK Solution and Cold Blood Cardioplegia on Systemic Endothelial Functions in Patients who Undergo Coronary Artery Bypass Surgery: a Prospective Randomized and Controlled Trial. Braz J Cardiovasc Surg. 2020;35(5). doi: 10.21470/1678-9741-2019-0327 PMC7598953 33118727

[16] Veres G, Radovits T, Merkely B, Karck M, Szabó G. Custodiol-N, the novel cardioplegic solution reduces ischemia/reperfusion injury after cardiopulmonary bypass. J Cardiothorac Surg. 2015;10(1). doi: 10.1186/s13019-015-0226-9 PMC4350983 25890005

[17] Schaefer M, Gebhard M-M, Gross W. The effect of melatonin on hearts in ischemia/reperfusion experiments without and with HTK cardioplegia. Bioelectrochemistry. 2019;129:170–8. doi: 10.1016/j.bioelechem.2019.05.017 31181439

[18] Chambers DJ. Mechanisms and alternative methods of achieving cardiac arrest polarized arrest an alternative to inducing arrest by depolarization (with elevated K concentrations) is to maintain polarization of. In: Asheville, NC.

[19] Yayla-Tunçer E, Şengelen A, Tan-Recep BZ, Şavluk ÖF, Yilmaz AA, Ceyran H, et al. Acute Changes in Myocardial Expression of Heat Shock Proteins and Apoptotic Response Following Blood, delNido, or Custodiol Cardioplegia in Infants Undergoing Open-Heart Surgery. Pediatr Cardiol. 2022;43(3):567–79. doi: 10.1007/s00246-021-02759-y 34694437

[20] Peng T, Zhang T, Lu X, Feng Q. JNK1/c-fos inhibits cardiomyocyte TNF-alpha expression via a negative crosstalk with ERK and p38 MAPK in endotoxaemia. Cardiovasc Res. 2009;81(4):733–41. doi: 10.1093/cvr/cvn336 19043087

[21] Sall A, Zhang HM, Qiu D, Liu Z, Yuan J, Liu Z, et al. Pro-apoptotic activity of mBNIP-21 depends on its BNIP-2 and Cdc42GAP homology (BCH) domain and is enhanced by coxsackievirus B3 infection. Cell Microbiol. 2010;12(5):599–614. doi: 10.1111/j.1462-5822.2009.01416.x 19951366

[22] Langendorff O. Untersuchungen am überlebenden Säugethierherzen. Pflügers Arch. 1895;61(6):291–332. doi: 10.1007/bf01812150

[23] Bell RM, Mocanu MM, Yellon DM. Retrograde heart perfusion: the Langendorff technique of isolated heart perfusion. J Mol Cell Cardiol. 2011;50(6):940–50. doi: 10.1016/j.yjmcc.2011.02.018 21385587

[24] Sato T, Takeda N. The roles of HIF-1α signaling in cardiovascular diseases. J Cardiol. 2023;81(2):202–8. doi: 10.1016/j.jjcc.2022.09.002 36127212

[25] Karimi M, Wang LX, Hammel JM, Mascio CE, Abdulhamid M, Barner EW, et al. Neonatal vulnerability to ischemia and reperfusion: cardioplegic arrest causes greater myocardial apoptosis in neonatal lambs than in mature lambs. The Journal of Thoracic and Cardiovascular Surgery. 2004;127(2):490–7. doi: 10.1016/j.jtcvs.2003.07.052 14762359

[26] Feng J, Bianchi C, Sandmeyer JL, Li J, Sellke FW. Molecular indices of apoptosis after intermittent blood and crystalloid cardioplegia. Circulation. 2005;112(9 Suppl):I184-9. doi: 10.1161/CIRCULATIONAHA.104.526160 16159813

[27] Livak KJ, Schmittgen TD. Analysis of relative gene expression data using real-time quantitative PCR and the 2(-Delta Delta C(T)) Method. Methods. 2001;25(4):402–8. doi: 10.1006/meth.2001.1262 11846609

[28] Dugbartey GJ. Cellular and molecular mechanisms of cell damage and cell death in ischemia-reperfusion injury in organ transplantation. Mol Biol Rep. 2024;51(1):473. doi: 10.1007/s11033-024-09261-7 38553658 PMC10980643

[29] Kale J, Osterlund EJ, Andrews DW. BCL-2 family proteins: changing partners in the dance towards death. Cell Death Differ. 2018;25(1):65–80. doi: 10.1038/cdd.2017.186 29149100 PMC5729540

[30] Wong DCP, Xiao J, Chew TW, Pan M, Lee CJM, Ang JW, et al. BNIP-2 Activation of Cellular Contractility Inactivates YAP for H9c2 Cardiomyoblast Differentiation. Adv Sci (Weinh). 2022;9(31):e2202834. doi: 10.1002/advs.202202834 35975420 PMC9631078

[31] Zhao Y, Xiong W, Li C, Zhao R, Lu H, Song S, et al. Hypoxia-induced signaling in the cardiovascular system: pathogenesis and therapeutic targets. Signal Transduct Target Ther. 2023;8(1):431. doi: 10.1038/s41392-023-01652-9 37981648 PMC10658171

[32] Kim J, Tchernyshyov I, Semenza GL, Dang CV. HIF-1-mediated expression of pyruvate dehydrogenase kinase: a metabolic switch required for cellular adaptation to hypoxia. Cell Metab. 2006;3(3):177–85. doi: 10.1016/j.cmet.2006.02.002 16517405

[33] Wu Z, Nicoll M, Ingham RJ. AP-1 family transcription factors: a diverse family of proteins that regulate varied cellular activities in classical hodgkin lymphoma and ALK+ ALCL. Exp Hematol Oncol. 2021;10(1):4. doi: 10.1186/s40164-020-00197-9 33413671 PMC7792353

[34] Chambers DJ, Fallouh HB. Cardioplegia and cardiac surgery: pharmacological arrest and cardioprotection during global ischemia and reperfusion. Pharmacol Ther. 2010;127(1):41–52. doi: 10.1016/j.pharmthera.2010.04.001 20398698

[35] Mishra P, Jadhav RB, Mohapatra CKR, Khandekar J, Raut C, Ammannaya GK, et al. Comparison of del Nido cardioplegia and St. Thomas Hospital solution - two types of cardioplegia in adult cardiac surgery. Kardiochir Torakochirurgia Pol. 2016;13(4):295–9. doi: 10.5114/kitp.2016.64867 28096823 PMC5233756

[36] Vittorini S, Storti S, Andreani G, Giusti L, Murzi B, Furfori P, et al. Heat shock protein 70-1 gene expression in pediatric heart surgery using blood cardioplegia. Clinical Chemical Laboratory Medicine. 2007;45(2). doi: 10.1515/cclm.2007.030 17311516

[37] Date T, Mochizuki S, Belanger AJ, Yamakawa M, Luo Z, Vincent KA, et al. Expression of constitutively stable hybrid hypoxia-inducible factor-1alpha protects cultured rat cardiomyocytes against simulated ischemia-reperfusion injury. Am J Physiol Cell Physiol. 2005;288(2):C314-20. doi: 10.1152/ajpcell.00374.2004 15496478

[38] Guimarães-Camboa N, Stowe J, Aneas I, Sakabe N, Cattaneo P, Henderson L, et al. HIF1α Represses Cell Stress Pathways to Allow Proliferation of Hypoxic Fetal Cardiomyocytes. Dev Cell. 2015;33(5):507–21. doi: 10.1016/j.devcel.2015.04.021 26028220 PMC4509618

[39] Wang F, Liang G-Y, Liu D-X, Tang Q, Zhang J, Cai Q-Y, et al. Effect of Si-RNA-silenced HIF-1α gene on myocardial ischemia-reperfusion-induced insulin resistance. Int J Clin Exp Med. 2015;8(9):15514–20. 26629042 PMC4658931

[40] Kang J-S, Bae G-U, Yi M-J, Yang Y-J, Oh J-E, Takaesu G, et al. A Cdo-Bnip-2-Cdc42 signaling pathway regulates p38alpha/beta MAPK activity and myogenic differentiation. J Cell Biol. 2008;182(3):497–507. doi: 10.1083/jcb.200801119 18678706 PMC2500135

[41] Zhang Y, Liu D, Hu H, Zhang P, Xie R, Cui W. HIF-1α/BNIP3 signaling pathway-induced-autophagy plays protective role during myocardial ischemia-reperfusion injury. Biomed Pharmacother. 2019;120:109464. doi: 10.1016/j.biopha.2019.109464 31590128

[42] Lu X, Lei M, Xiang F, Feng Q. Abstract 162: c-fos Protects the Myocardium from Ischemic Injury and Improves Cardiac Function. Circulation. 2007;116(suppl_16). doi: 10.1161/circ.116.suppl_16.ii_10-a

[43] Bao Y, Qiao Y, Yu H, Zhang Z, Yang H, Xin X, et al. miRNA-27a Transcription Activated by c-Fos Regulates Myocardial Ischemia-Reperfusion Injury by Targeting ATAD3a. Oxid Med Cell Longev. 2021;2021:2514947. doi: 10.1155/2021/2514947 34413925 PMC8369174

[44] Poetsch MS, Strano A, Guan K. Role of Leptin in Cardiovascular Diseases. Front Endocrinol. 2020;11. doi: 10.3389/fendo.2020.00354 PMC7325922 32655492

[45] Genoux A, Bastard J-P, groupe de travail RIHN Adipokines. Effects of leptin and adiponectin on the cardiovascular system. Ann Biol Clin (Paris). 2020;78(3):253–60. doi: 10.1684/abc.2020.1548 32420888

[46] Gorjipour F, Dehaki MG, Totonchi Z, Hajimiresmaiel SJ, Azarfarin R, Pazoki-Toroudi H, et al. Inflammatory cytokine response and cardiac troponin I changes in cardiopulmonary bypass using two cardioplegia solutions; del Nido and modified St. Thomas’: a randomized controlled trial. Perfusion. 2017;32(5):394–402. doi: 10.1177/0267659117691119 28152655

