# Supplementary material for: How different cardioplegic solutions influence genes expression and cytokine response in an immature rat heart model of ischemia/reperfusion?
Source: PLoS One. 2025 Jul 29;20(7):e0329010. doi: 10.1371/journal.pone.0329010 (PMC12306747; doi:10.1371/journal.pone.0329010)
Supplement: S1 Table — (PDF) [file pone.0329010.s001.pdf]

Table S1. HIF1α ΔCt by solution and ischemia duration

Solution	Time (h)	Mean HIF1α (ΔCt)	Std Dev
ST	1	2.36	0.22
ST	2	2.22	0.22
ST	4	2.29	0.31
HTK	1	2.18	0.11
HTK	2	1.64	0.16
HTK	4	1.84	0.34
DN	1	1.41	0.32
DN	2	1.13	0.31
DN	4	1.52	0.74
