# Supplementary material for: How different cardioplegic solutions influence genes expression and cytokine response in an immature rat heart model of ischemia/reperfusion?
Source: PLoS One. 2025 Jul 29;20(7):e0329010. doi: 10.1371/journal.pone.0329010 (PMC12306747; doi:10.1371/journal.pone.0329010)
Supplement: S2 Table — (PDF) [file pone.0329010.s002.pdf]

Table S2. FOS ΔCt by solution and ischemia duration

Solution	Time (h)	Mean FOS (ΔCt)	Std Dev
ST	1	-4.59	0.22
ST	2	-4.60	0.55
ST	4	-4.80	0.42
HTK	1	-4.28	0.50
HTK	2	-4.64	0.93
HTK	4	-4.93	1.03
DN	1	-5.38	1.33
DN	2	-6.93	0.86
DN	4	-6.52	1.17
