# Supplementary material for: How different cardioplegic solutions influence genes expression and cytokine response in an immature rat heart model of ischemia/reperfusion?
Source: PLoS One. 2025 Jul 29;20(7):e0329010. doi: 10.1371/journal.pone.0329010 (PMC12306747; doi:10.1371/journal.pone.0329010)
Supplement: S3 Table — (PDF) [file pone.0329010.s003.pdf]

Table S3. BNIP2 ΔCt by solution and ischemia duration

Solution	Time (h)	Mean BNIP2 (ΔCt)	Std Dev
ST	1	1.38	0.21
ST	2	1.51	0.16
ST	4	1.82	0.54
HTK	1	1.47	0.24
HTK	2	0.96	0.27
HTK	4	1.18	0.56
DN	1	0.48	0.88
DN	2	0.37	0.51
DN	4	0.75	1.10
