# Supplementary material for: How different cardioplegic solutions influence genes expression and cytokine response in an immature rat heart model of ischemia/reperfusion?
Source: PLoS One. 2025 Jul 29;20(7):e0329010. doi: 10.1371/journal.pone.0329010 (PMC12306747; doi:10.1371/journal.pone.0329010)
Supplement: S4 Table — (PDF) [file pone.0329010.s004.pdf]

Table S4. BAX/BCL2 Ratio by solution and ischemia duration

Solution	Time (h)	Mean BAX/BCL2 Ratio	Std Dev
ST	1	0.79	0.12
ST	2	0.80	0.15
ST	4	0.94	0.12
HTK	1	0.86	0.13
HTK	2	0.89	0.17
HTK	4	0.94	0.14
DN	1	0.56	0.18
DN	2	0.46	0.10
DN	4	0.65	0.17
