# Supplementary material for: How different cardioplegic solutions influence genes expression and cytokine response in an immature rat heart model of ischemia/reperfusion?
Source: PLoS One. 2025 Jul 29;20(7):e0329010. doi: 10.1371/journal.pone.0329010 (PMC12306747; doi:10.1371/journal.pone.0329010)
Supplement: S5 Table — (PDF) [file pone.0329010.s005.pdf]

Table S5. IL-4 Levels by Solution and Ischemia Duration

Solution	Time (h)	Mean IL-4 (pg/mL)	Std Dev
ST	1	5.82	2.29
ST	2	7.36	4.48
ST	4	4.12	2.96
HTK	1	5.12	5.32
HTK	2	4.01	1.30
HTK	4	3.51	2.28
DN	1	4.77	2.31
DN	2	3.25	1.95
DN	4	2.78	2.16
