# Supplementary material for: How different cardioplegic solutions influence genes expression and cytokine response in an immature rat heart model of ischemia/reperfusion?
Source: PLoS One. 2025 Jul 29;20(7):e0329010. doi: 10.1371/journal.pone.0329010 (PMC12306747; doi:10.1371/journal.pone.0329010)
Supplement: S6 Table — (PDF) [file pone.0329010.s006.pdf]

Table S6. IL-6 levels by solution and ischemia duration

Solution	Time (h)	Mean IL-6 (pg/mL)	Std Dev
ST	1	16.93	7.73
ST	2	24.09	18.22
ST	4	19.38	14.98
HTK	1	15.48	5.94
HTK	2	15.25	7.10
HTK	4	15.79	10.29
DN	1	17.11	9.32
DN	2	16.08	12.09
DN	4	14.53	9.29
