# Supplementary material for: How different cardioplegic solutions influence genes expression and cytokine response in an immature rat heart model of ischemia/reperfusion?
Source: PLoS One. 2025 Jul 29;20(7):e0329010. doi: 10.1371/journal.pone.0329010 (PMC12306747; doi:10.1371/journal.pone.0329010)
Supplement: S7 Table — (PDF) [file pone.0329010.s007.pdf]

Table S7. IL-10 levels by solution and ischemia duration

Solution	Time (h)	Mean IL-10 (pg/mL)	Std Dev
ST	1	52.31	19.22
ST	2	57.34	30.08
ST	4	49.45	16.55
HTK	1	50.32	13.87
HTK	2	54.05	12.71
HTK	4	54.09	19.97
DN	1	58.41	22.41
DN	2	58.25	15.11
DN	4	52.33	13.18
