# Supplementary material for: How different cardioplegic solutions influence genes expression and cytokine response in an immature rat heart model of ischemia/reperfusion?
Source: PLoS One. 2025 Jul 29;20(7):e0329010. doi: 10.1371/journal.pone.0329010 (PMC12306747; doi:10.1371/journal.pone.0329010)
Supplement: S8 Table — (PDF) [file pone.0329010.s008.pdf]

Table S8. Leptin levels by solution and ischemia duration

Solution	Time (h)	Mean Leptin (pg/mL)	Std Dev
ST	1	25.20	6.73
ST	2	22.83	6.28
ST	4	19.45	6.12
HTK	1	22.56	5.36
HTK	2	18.44	6.66
HTK	4	19.80	7.29
DN	1	23.13	7.26
DN	2	20.23	7.33
DN	4	17.37	5.33
