# Supplementary material for: How different cardioplegic solutions influence genes expression and cytokine response in an immature rat heart model of ischemia/reperfusion?
Source: PLoS One. 2025 Jul 29;20(7):e0329010. doi: 10.1371/journal.pone.0329010 (PMC12306747; doi:10.1371/journal.pone.0329010)
Supplement: S9 Table — (PDF) [file pone.0329010.s009.pdf]

Table S9. TNF-α levels by solution and ischemia duration

Solution	Time (h)	Mean TNF-α (pg/mL)	Std Dev
ST	1	7.52	2.17
ST	2	7.28	2.30
ST	4	6.46	2.20
HTK	1	7.84	1.80
HTK	2	7.52	2.56
HTK	4	7.58	2.21
DN	1	7.60	2.27
DN	2	7.57	1.90
DN	4	6.93	2.18
